# An affordable open-source hydrophone for low-frequency underwater acoustic measurements for educational and small-laboratory applications

**DOI:** 10.1016/j.ohx.2026.e00798

**Published:** 2026-05-24

**Authors:** Nhut Thang Le, Ngoc Hung Nguyen, Cong Toai Truong, Huy Hung Nguyen, Tan Tien Nguyen, Van Tu Duong

**Affiliations:** a Key Laboratory of Digital Control and System Engineering (DCSELab), Faculty of Mechanical Engineering, Ho Chi Minh University of Technology (HCMUT), 268 Ly Thuong Kiet Street, Dien Hong Ward, Ho Chi Minh City, Viet Nam; b Vietnam National University Ho Chi Minh City, Linh Xuan Ward, Ho Chi Minh City, Viet Nam; c Faculty of Electrical and Electronics Engineering, Ho Chi Minh City University of Technology and Engineering, Ho Chi Minh City, Vietnam

**Keywords:** Infrasonic hydrophone, Piezoelectric underwater sensor, 3D printing, Low-cost, Open-source hardware

## Abstract

Infrasonic underwater acoustic signals (below 20 Hz) are essential for environmental monitoring, marine ecosystem studies, and evaluation of underwater robotic systems. Nevertheless, existing commercial hydrophones are primarily designed for broadband and high-frequency performance, increasing complexity and cost while limiting ultra-low-frequency stability. Additionally, rapid-prototyping hydrophone designs depend on available modules and lack standardized design guidelines, limiting further modifications. As a result, this research presents a low-cost, open-source infrasonic hydrophone (LUH) for underwater measurements, compact and portable with approximately 500 g, with a modular configuration fabricated by acrylonitrile butadiene styrene (ABS) using fused deposition modelling (FDM)- based 3D printing for rapid prototyping. Waterproofing, noise suppression, and signal transmission are achieved through multiple layers of elastic dip, copper foil, and silicone. From an electrical perspective, the LUH integrates a piezoelectric ceramic disk, high-impedance amplification, band-limited filtering, and a modular ± 12 VDC power supply within enclosures. The complete device can be fabricated within 100 h at a total cost of below 90 USD. The experimental validation demonstrates the stable detection of a 20 Hz sinusoidal signal under laboratory conditions, the 12 Hz accurate measurement using institutional-grade equipment, and the detection of a 5 Hz emission from a biomimetic fish robot at 3 m underwater. From there, these results have confirmed the reliable infrasonic sensing ability of the device in various situations.

## Graphical abstract

**Figure:**
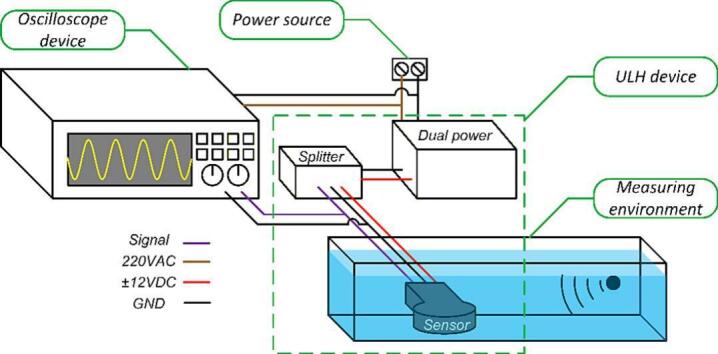

Specifications table

**Table:** 

Hardware name	*Low-frequency underwater hydrophone (LUH)*
Subject area	• Engineering and materials science • Educational tools and open-source alternatives to existing infrastructure • General
Hardware type	• Measuring physical properties and in-lab sensors • Field measurements and sensors • Mechanical engineering and materials science
Closest commercial analog	Type 8106 HydrophoneTeledyne Marine Reference Hydrophone SeriesCR-Series Hydrophone Systems
Open-source license	CC BY 4.0
Cost of hardware	Under $90
Source file repository	https://doi.org/10.17632/42mksnzmw2.2

## 1 Hardware in context

In the era of rapid industrialization, technological advancements, particularly in marine science, underwater technology, and environmental science, have significantly expanded human activities in underwater environments. However, these activities are highly sensitive to acoustic disturbances, as noise can strongly affect both system performance and environmental conditions [1], [2]. Therefore, the study and monitoring of underwater acoustic phenomena have become increasingly important for both research and educational purposes. Indeed, the study on underwater acoustic waves, such as low-frequency acoustic waves lower than 20 Hz, has recently attracted much attention in a variety of scientific and engineering fields [3], [4]. Despite the fact that these waves are not audible to the human ear, they play an important role in determining physical processes and influencing biological behaviors in aquatic systems [5], [6]. The ability to track the responses to these waves can aid in the achievement of significant goals such as the exploration of the ocean, the development of technologies in the water, and education. By tracking specific waves and understanding the communication behaviors among aquatic organisms, scientists and engineers can obtain greater insights into the aquatic ecosystem through quantified and visualized representations [7], [8]. In light of these considerations, the design of unmanned underwater systems can be made more environmentally sustainable by monitoring acoustic emissions during system operation and minimizing noise-induced ecological impacts [9]. Given this potential, underwater acoustic measurement systems are not only capable of detecting infrasonic signals but also are accessible for widespread adoption, particularly within educational institutions and research communities [10]. Accordingly, there has been an increasing need to have cost-effective, as well as easy-to-fabricate, hydroacoustic devices that have the ability to detect as well as visualize infrasonic underwater signals. This calls for an “impressive need” to have accessible measurement systems, which are specifically designed to be used in low-frequency acoustic applications.

There have been many commercial products and research efforts developed to detect and process infrasonic waves underwater. One of the products that can be used to measure infrasonic waves is the reference hydrophone series from Teledyne Marine, which provides a full range of high-performance hydrophones and transducers that meet the harsh requirements of underwater acoustic applications. The product can measure frequencies from 1 Hz to 480 kHz [11]. Nevertheless, the major drawback of these sensors lies in their high cost, which varies from USD 450 referencing from the commercial markets and Vietnam distributors, depending on the frequency range selection, depth rating, and calibration services. Another research was carried out by the CR-series hydrophones, which were designed by the Cetacean Research Technology (CRT). These sensors are commonly used in commercial whale-watching operations [12]. These systems are user-friendly and deliver high-quality audio, which allows the identification of vocalizations of whales, dolphins, and porpoises within the human hearing spectrum. Although the basic kit, together with a Zoom H1n device, costs around USD 200 referencing from the commercial markets and Vietnam distributors, which can be used by a novice, the total cost of a complete system, which would be used by a researcher, would be higher, as it would require the addition of a dedicated device, a speaker, a waterproof case, as well as specialized software such as SpectraPLUS-SC, which would amount to around USD 2400. In Vietnam, international products are distributed by companies such as Brüel & Kjær [13], which provides the low-noise hydrophone type 8106 with the ability to measure frequencies between 7 Hz and 80 kHz. However, effective use of Brüel & Kjær hydrophones requires other specific measurement instrumentation to be purchased, and the costs for these measurement solutions provided by the company are substantially high. Apart from the constraints related to the costs associated with the use of these hydrophones, the primary disadvantage of these hydrophones is their ability to measure across a broad frequency range, rather than specifically within the range required for underwater infrasound [14]. With regards to measurement, the broadband capability creates inherent trade-offs in sensor sensitivity, gain allocation, and mechanical isolation [15]. For the case of wideband hydrophones, the amplification and dynamic range allocation in the front end are set to prevent saturation at higher frequency bands. As such, the sensitivity and signal-to-noise ratio are compromised in the ultra-low frequency band. To reduce costs and speed up the experimental process, there is an alternative solution in the form of rapid prototyping hydrophones for educational and cost-effective experiments [16]. These types of prototypes usually require the availability of different components, such as piezoelectric elements, operational amplifier modules, as well as general enclosures. Nevertheless, the main problem that is usually observed is the lack of stability, as the quality of the waterproofing as well as the mechanical assembly may be unsatisfactory. Additionally, the implementation of the prototype fails to come with the availability of standardized guidelines, as the availability of the electronic module is difficult to modify, thus providing the user with limited options for amplification as well as filter tuning for the infrasonic range.

In summary, there are a number of key issues that continue to prevent the widespread use of low-frequency underwater acoustic measurement devices. Firstly, the cost of available solutions remains prohibitively expensive for use in educational institutions or by small research groups. Secondly, the available solutions focus on broadband frequency response rather than infrasonic response, which means that the sensitivity of the device to the frequency of interest remains too low, as well as the signal-to-noise ratio. Thirdly, low-cost solutions exhibit low stability, which is insufficient for use in low-frequency underwater sensing. Last but not least, there remains a lack of detailed design guidelines, which has hindered the widespread use of low-cost underwater acoustic measurement solutions.

To bridge the identified gap, this paper proposes the development of an affordable waterproof hydrophone device, referred to as “Low-frequency underwater hydrophone” (LUH), which consists of the sensor head module together with analog conditioning, the 3D enclosures as well as modular interconnection. The proposed device is intended for sub-20 Hz underwater acoustic measurements. Specifically, the proposed device aims to provide an affordable solution for educational institutions and small research laboratories to conduct underwater acoustic measurements, as existing commercial solutions are too expensive for such environments. A total device cost of less than USD 90 was set as the objective to ensure the proposed device is highly accessible and reproducible. The proposed hydrophone device includes low-cost piezoelectric sensing elements and amplification and filtering stages to ensure effective underwater operation. Furthermore, the proposed device is designed to prioritize performance in the infrasound range to improve the signal-to-noise ratio at ultra-low frequencies. Importantly, to improve the reproducibility and educational value of the proposed device, the paper presents a design methodology including a step-by-step electrical design guidance and mechanical design that requires approximately 100 h to develop the device. In consequence, the proposed hydrophone device becomes an effective tool for research on underwater acoustics. Markedly, for low frequencies, it becomes an effective tool for hands-on instruction in acoustics and instrumentation design in engineering programs.

## 2 Hardware description

The LUH is a cost-efficient frequency-measuring solution designed to record low-frequency underwater signals with a range below 20 Hz. With its modular, compact design, the LUH aims to serve multiple applications in education and related fields of biomimetics. The water-resistant acrylonitrile butadiene styrene (ABS) via fused deposition modeling (FDM) is used for manufacturing the sensor enclosures, the LUH leverages 3D printing’s cost efficiency and rapid prototyping flexibility. In order to simplify the design as well as enhance the manufacturing time, the idea of modularization for the LUH is conducted by three main modules comprising a sensor head module, a dual power supply module, and a signal splitter module. Simultaneously, the complete mechanical parts include a sensor base and a sensor coverage with a total printing time of approximately 10 h. Besides, the signal splitter box and the power box are based on available commercial products with low prices. In general, the total weight of the LUH is approximately 500 g, and it occupies a footprint of (200x150x100) mm. Based on the design, the mechanical assembly mainly utilizes screwing installation, making it compact and portable for deployment in constrained laboratory spaces and classroom settings.

The most important things in designing the hydrophone devices are noise filtering and water resistance. Due to the fact that noise is introduced mechanically before the signal exposes the sensing component, a noise suppression approach using a multi-layer mechanism and a material-based filtering strategy is proposed. The first layer is applied to the inner surface of the sensor base, where a continuous copper foil lining is applied. This copper layer serves as an electromagnetic shield, forming a closed, conductive enclosure around the piezoelectric chamber. The thickness of the copper layer is chosen based on the principle of the Faraday cage [17], which yields the sheet resistance of the copper foil:

(1) $$R_{s}=\frac{\rho_{\mathrm{Cu}}}{t_{\mathrm{Cu}}},$$

where $R_{s}$ is the sheet resistance of the copper foil (Ω), $\rho_{\mathrm{Cu}}$ is the coil’s electrical resistivity (Ωm) with $\rho_{\mathrm{Cu}}=1.68\times 10^{-8}\Omega m$, and $t_{\mathrm{Cu}}$ is the copper layer’s thickness (m). From the Eq. (1) with the chosen commercial copper foil thickness of 0.1 mm, the sheet resistance is equal to $1.68\times 10^{-4}\Omega$, this extremely small sheet resistance helps charge distribute uniformly across its surface, creating an equipotential enclosure. In addition, with small resistance, there is no high potential gradient across the shield’s surface, leading to no external noise coupling into the internal components.

The two remaining layers, including the silicone layer and the elastic dip layer, are applied to perform mechanical noise suppression and waterproofing. To determine the material and the thickness of layers, according to [18], the two fundamental acoustic characteristics are considered: efficient transmission of pressure waves across material interfaces and the natural frequency of the propagating acoustic signal. Upon encountering an acoustic wave encounters a boundary between two media, the transmission efficiency depends on the impedance contrast, where acoustic impedance is defined as

(2) Z=ρc,

where Z is the acoustic impedance of the medium $\left(kg/m^{2}s\right)$, c is the acoustic wave velocity (m/s) in that medium, and ρ is the material density $\left(kg/m^{3}\right)$ yielded by

(3) $$\rho =\frac{m}{V},$$

where m is the mass of the material (kg) and V is the volume of the material $\left(m^{3}\right)$, which is proportional to the thickness. Based on Eq. (2) and Eq. (3), the change of the impedance can depend on the material thickness. The larger impedance mismatches between two media are, the more significant the reflection result from, whereas materials whose impedance is closer to that of water enable more efficient pressure transmission. On the other hand, in order to ensure the signal transmission, the key principle is that the frequency of the signal must be kept the same. Based on this, the natural frequency of the material, which allows the material to oscillate without the dependence on external force, is determined based on the mass-spring-damper model

(4) $$f_{n}=\frac{1}{2\pi }\sqrt{\frac{k}{m_{\mathrm{eq}}}},$$

where $f_{n}$ is the natural frequency of the material system $\left(Hz\right)$, $m_{\mathrm{eq}}$ is the mass of the piezo and the silicone (kg), and k is the stiffness of the silicone (N/m) presented by

(5) $$k=\frac{\mathrm{EA}}{t},$$

where E is the Young’s modulus of the silicone $\left(N/m^{2}\right)$, A is the area of the silicone layer ($m^{2}$), and t is the thickness of the silicone layer (m). Following the Eq. (4) and Eq. (5), the natural frequency of the material can be adjusted due to the thickness. With the two key characteristics of acoustic transmission, the trade-off dependent on the material thickness is noted that the larger the thickness is, the lower the natural frequency of the material can be achieved; nevertheless, the impedance of the material is considered to avoid the increasing mismatch between the impedances of the material and the environment. Within this framework, the selection of layer thickness is guided by the requirement to maintain sufficient acoustic coupling with water while enabling the sensing structure to respond effectively in the infrasonic range (<20 Hz). The final thickness values of 14 mm for the silicone layer and 0.5 mm for the elastic dip coating were determined through iterative experimental tuning, informed by the material properties reported in “Tekbond Axit Acetoxy”[19]. These layers are built by multiple layers so that they can be uniform and continuous to form a moisture barrier as well as isolate the inside electric components from water exposure. In addition, it is possible to substitute the LUH’s materials with alternative materials, which share the same characteristics of Young’s modulus factor and material density. Simultaneously, the alternative materials need to achieve low acoustic impedance to minimize mismatch with the surrounding environment, as well as a suitable thickness to maintain the signal transmission. Notably, the materials are required to be capable of forming uniform and continuous layers to serve as a moisture barrier.

**Fig. 1:**
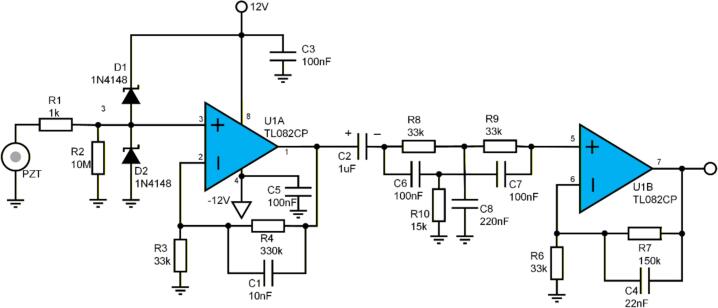
Schematic circuit design.

From an electrical perspective, the main power source that supplies the device is the alternating current (AC) voltage of 220 VAC – 50/60 Hz. The power source is then converted to direct current (DC) at ± 12 VDC to power the sensor unit and support system expansion. In addition, the sensor’s output signal is read using external readout equipment. In the proposed amplifier board design shown in Fig. 1, the PZT sensor is represented as an ideal voltage source in series with its intrinsic capacitance. A high-value input resistor of approximately 10 MΩ is employed at the front end to provide a return path for DC and to define the operating point. This selection is based on the electrical characteristics of the piezoelectric ceramic disk, as the sensor can be modeled as a voltage source in series with its intrinsic capacitance in the nano-farad range. This behavior results in a high source impedance at low frequencies; therefore, the input resistance of the amplifier must be sufficiently large to avoid signal attenuation. In the proposed design, the front-end uses a 10 MΩ input resistor together with an operational amplifier that has low input bias current, which establishes an input impedance in the megaohm range. This high input impedance minimizes loading of the sensor, and as a result the signal amplitude is preserved in the low-frequency region. At the same time, the input resistor forms a high-pass response with the sensor capacitance, where the cutoff frequency is given by $f_{c}=1/2\pi RC$. For capacitance values of $\left[10,100\right]$ nF, the cutoff frequency ranges from approximately 0.16 Hz to 1.6 Hz, which lies well below the target measurement band below 20 Hz. Therefore, the selected value of 10 MΩ is adequate for low-frequency signal acquisition and demonstrates that the circuit operates as a high-input-impedance amplification stage. Following the input stage, the signal is first processed by a non-inverting operational amplifier that provides amplification and initial frequency shaping, with a low-frequency gain of approximately 11. In this stage, a feedback capacitor is connected in parallel with the feedback resistor, which introduces a low-pass characteristic. As a result, a dominant pole is formed at approximately 48 Hz, leading to limited high-frequency amplification and reduces noise. Subsequently, the output of the first stage is connected to the second stage through an AC coupling capacitor of approximately 1 µF. In this configuration, the coupling capacitor forms a high-pass response with the input resistance of the second stage in the order of tens of kilo-ohms, and the resulting cutoff frequency is approximately 3 Hz. Therefore, this stage suppresses DC components and very low-frequency drift while preserving the infrasonic signal content of interest. Besides, the signal is further processed by the second non-inverting operational amplifier, which provides a gain of approximately 5.5. Accordingly, a feedback capacitor is also implemented in this stage to introduce a low-pass characteristic similar to the first stage, thereby maintaining the upper frequency limit around 48 Hz. Moreover, an additional passive RC network is included to further enhance the attenuation of higher-frequency components and stabilize the frequency response. Briefly, the overall gain reaches approximately 60 as the signal passes through both amplification stages. At the same time, the high-pass response at approximately 3 Hz and the low-pass response at approximately 48 Hz define the effective operating band, which matches the infrasonic measurement requirement and suppresses DC drift and high-frequency noises. To explain the design of the circuit, the electric design guideline file “LUH_Instruction_Electric circuit design” has been added as supplementary material to the LUH_Instruction.

As a result, the contribution of the hardware LUH consists of:

• Applying a 3D-printed enclosure and using the multi-layer waterproof and noise filtering mechanism enables low-cost and rapid manufacturing.
• Modular and compact layout simplifies installation and operation, making the device suitable for various research and education applications.
• PCB mainboard integrating high-impedance amplification and band-limiting functions allows direct control over noise performance and frequency response.
• Expandable via standardized power and signal connectors, enabling the integration of additional sensor heads or auxiliary modules for multi-point hydroacoustic measurements.

## 3 Design files summary

LUH_Schematic: comprises the schematic design of LUH for the main control board to be used in the drawing of the PCB. It specifies component interconnections, signal routing, power distribution, supporting device control, and peripheral communication (Table 1).

**Table 1:** List of components of the LUH.

Design file name	File type	Open-source license	Location of the file
LUH_Schematic	PDF	CC BY 4.0	https://doi.org/10.17632/42mksnzmw2.2
LUH_Project	ZIP	CC BY 4.0	https://doi.org/10.17632/42mksnzmw2.2
LUH_Assem	ZIP	CC BY 4.0	https://doi.org/10.17632/42mksnzmw2.2
LUH_Instruction	PDF	CC BY 4.0	https://doi.org/10.17632/42mksnzmw2.2
LUH_Experiment	ZIP	CC BY 4.0	https://doi.org/10.17632/42mksnzmw2.2
LUH_Electrical BOM	xlsx	CC BY 4.0	https://doi.org/10.17632/42mksnzmw2.2
LUH_Mechanical BOM	xlsx	CC BY 4.0	https://doi.org/10.17632/42mksnzmw2.2

LUH_Project: entire Altium Designer project for the LUH device’s main controller’s schematic and multi-layer PCB. This includes the functional structure for the PCB, component placement, track routing, as well as the layers for the PCB for signal integrity during operation.

LUH_Assem: contains the 3D mechanical assembly for the LUH device, which includes the PCB, mechanical enclosures, etc. This ensures compatibility as well as visualization during assembly of the entire device.

LUH_Instruction: provides a step-by-step guide detailing the calculations and methodologies used to design the electrical parts of the LUH, which is compatible with the LUH_Schematic. Its primary purpose is to ensure that the electronic design meets the target frequency measurement bandwidth with optimal signal-to-noise ratio (SNR) and proper impedance matching. Besides, it provides experimental records that demonstrate full experimental procedures.

LUH_Experiment: offers three experimental videos that validate the results obtained in Section 7 of the paper. The purpose of this package is to provide practical evidence of the experiment’s results, which was discussed in the paper.

LUH_Electrical BOM: This contains the listing of all the electrical components associated with LUH_Schematic, along with their designators, component details, quantity, cost per unit, and their sourcing.

LUH_Mechanical BOM: It includes a detailed list of all mechanical parts that are associated with LUH_Assem. The information that is included is designators, component descriptions, quantities, material Types, and sourcing Information.

## 4 Bill of materials summary

This section presents a summary of the materials used in the LUH system. More specifically, the bill of materials (BOM) is divided into two parts, comprising Table 2, Table 3, which list the electrical BOM and mechanical BOM, respectively. As a result, these tables provide a clear overview of the components and materials needed to reproduce the LUH system.

**Table 2:** Bill of electrical materials.

Designator	Component	Number	Cost per unit −currency	Total cost −currency	Source of materials	Material type
C1	10nF 50 V Capacitor	1	$0.75	$0.75	https://mou.sr/4cu8lYu	Semiconductor/others
C2	1uF 50 V Capacitor	1	$1.26	$1.26	https://mou.sr/3MAENhu	Semiconductor/others
C3, C5, C6, C7	100nF 50 V Capacitor	4	$0.37	$1.48	https://mou.sr/3OlaFXP	Semiconductor/others
C4	22nF 50 V Capacitor	1	$1.4	$1.4	https://mou.sr/4awPYQj	Semiconductor/others
C8	220nF 50 V Capacitor	1	$0.36	$0.36	https://mou.sr/4tLbNEC	Semiconductor/others
D2, D1	1N4148W	2	$0.1	$0.2	https://mou.sr/4kzqWVh	Semiconductor/others
J1	Header 2 pin	1	$0.49	$0.49	https://mou.sr/3MEATnL	Semiconductor/others
J2	Header 4 pin	1	$0.66	$0.66	https://mou.sr/3O2Rke6	Semiconductor/othSemiconductor/othersers
R1	SM-RES-0805 1 K	1	$0.33	$0.33	https://mou.sr/408OMxu	Semiconductor/othSemiconductor/othersers
R2	SM-RES-0805 10 M	1	$0.1	$0.1	https://mou.sr/4twuifG	Semiconductor/othSemiconductor/othersers
R3, R6, R8, R9	SM-RES-0805 33 K	4	$0.23	$0.92	https://mou.sr/4cno1wE	Semiconductor/othSemiconductor/othersers
R4	SM-RES-0805 330 K	1	$0.1	$0.1	https://mou.sr/4rOIWxm	Semiconductor/othSemiconductor/othersers
R7	SM-RES-0805 150 K	1	$0.16	$0.16	https://mou.sr/4cpkRbL	Semiconductor/othSemiconductor/othersers
R10	SM-RES-0805 15 K	1	$0.19	$0.19	https://mou.sr/4kw4Sec	Semiconductor/othSemiconductor/othersers
U1	TL082CP	1	$0.97	$0.97	https://mou.sr/4qrRSHX	Semiconductor/othSemiconductor/othersers

**Table 3:** Bill of mechanical materials.

Designator	Component	Number	Cost per unit −currency	Total cost −currency	Source of materials	Material type
HY11003	Main board	1	$3	$3	[Custom Supplier]	−Other
HY11004	Button Head Hex-Drive Screws, M3x10mm	16	$0.22	$3.52	https://www.amazon.com/M3x10mm-Thread-Stainless-Socket-DIN912/dp/B07FT6FBHP?currency = USD	−Metal
HY11009	Socket head screw M3x10mm	4	$0.19	$0.96	https://www.alibaba.com/product-detail/Stainless-Steel-Din-912-A2-70_1600912663102.html	−Metal
HY11005	Piezoelectric ceramic	1	$0.28	$0.28	https://www.alibaba.com/product-detail/Copper-Piezo-Buzzer-Piezoelectric-Element-Ceramic_1600864503441.html	−Brass
HY11008	Sensor coverage	1	$3.84	$3.84	[Custom Supplier]	−Polymer
HY11007	Electrode	1	$0.2	3,85	[Custom Supplier]	−Copper
HY11010	Multi-core cable	5	$0.23	$1.15	https://www.amazon.co.uk/Mercury-808-201UK-Core-Alarm-Cable-WHITE/dp/B016IXLJQQ?th = 1	−Polymer, Copper.
HY12006	Cable strain relief	1	$3.63	$3.63	[Custom Supplier]	−Polymer
HY13001	Sensor base	1	$12.5	$12.5	[Custom Supplier]	−Polymer
HY11012	Tekbond Axit Acetoxy	1	$2.7	$2.7	https://www.saint-gobain-africa.com/en/tekbond	−Silicone
HY11013	Elastic dip	1	$6.36	$6.36	https://www.bosny.com/product/elastic-spray-paint-b126/	−Rubber
HY11014	Micro M12 Screw-TogetherConnectors – 3 Pins	5	$2.12	$10.60	https://www.alibaba.com/product-detail/AOHUA-M14-Outdoor-Cable-Waterproof-Electrical_1600052904816.html	−Metal
HY11015	Switching power source – 12VDC/4A	2	$7.70	$15.40	https://www.alibaba.com/product-detail/Adjustable-D-30 W-5 V-4A-12V_11000017154793.html	−Metal
HY11016	Fan 12VDC	1	$3.85	$3.85	https://www.alibaba.com/product-detail/Hot-Selling-5015M12S-Computer-Chip_1601669887791.html	−Polymer
HY11017	Quick Connection Terminal Three-in / Nine-out	1	$0.65	$0.65	https://www.alibaba.com/product-detail/Household-Appliance-Copper-Durable-Quick-Connection_1601629247564.html	−Polymer
HY11018	Splitter module box	1	$6.16	$6.16	https://www.alibaba.com/product-detail/150–100–80 mm-IP66-Middle-Customized_1601200111496.html	−Polymer
HY11019	Power supply module box	1	$3.08	$3.08	https://www.alibaba.com/product-detail/265–185–95-Waterproof-Plastic-Electronic_1601224505118.html	−Polymer

## 5 Build instructions

### 5.1 Overall system description

**Fig. 2:**
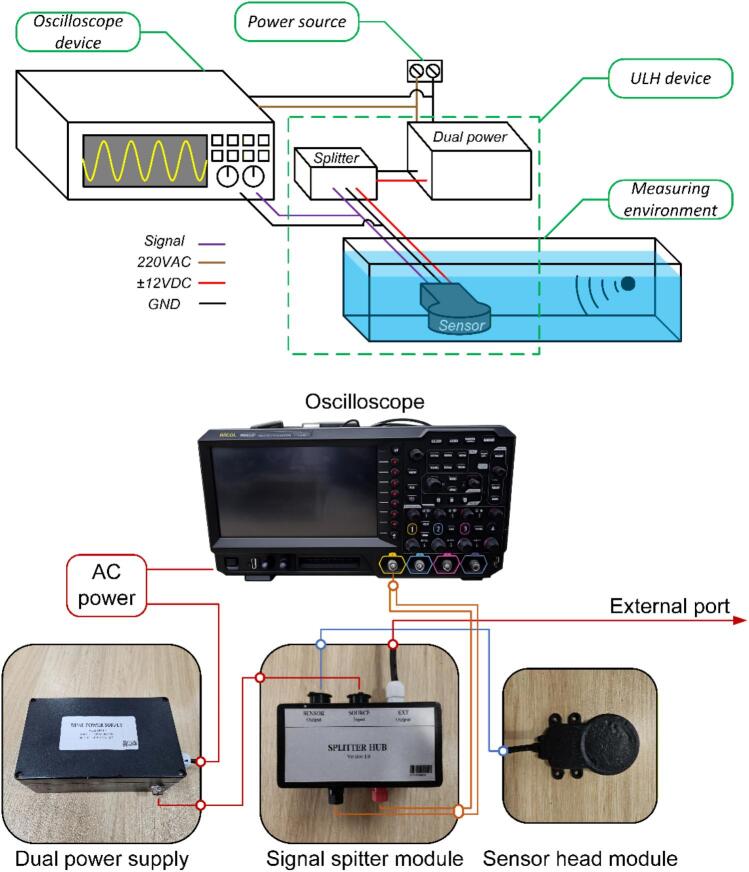
Schematic overview of the LUH system.

Fig. 2 illustrates a schematic overview of the LUH system, which includes proposed hardware and further external devices to acquire and analyse the signal: (1) the proposed LUH device – (i) the sensor head module, (ii) the dual power supply and (iii) the signal splitter; as well as (2) the external equipment – (i) power source; (ii) the oscilloscope device; and (iii) measuring environment. The LUH’s sensor head module is placed in the measuring environment near the sound source in order to record the oscillating frequency. Additionally, these modules are connected by four lines with different gauges for signal and power: the brown line supplies the dual power supply module, which converts AC power to ± 12 VDC DC power for the signal splitter and sensor head, as illustrated by the red and black lines, respectively. On the other hand, the purple lines represent the signal wires for communication between the signal splitter module, the sensor head module, and the oscilloscope device. In detail, according to the three mechanical modules (i), (ii), (iii), the electrical components are structured into them to perform the corresponding functions. Firstly, the dual power supply module regulates the input voltage from the source, which is in the form of an alternating current (AC) voltage and is measured to be 220 VAC at 50/60 Hz. This voltage is then converted to a regulated DC voltage, which is measured to be ± 12 VDC. This is achieved through the use of two isolated switch-mode power supplies (SMPS), which are connected in a complementary manner to achieve the desired output voltage of ± 12 VDC, which is symmetrical and has the same reference ground. The output lines from the dual power supply module, such as + 12 VDC, −12 VDC, and GND, are connected to the cooling fan located in the enclosure of the module to achieve thermal dissipation. In parallel, the power supply lines are routed to the “SOURCE input” port of the signal splitter module as the primary power source to the electrical devices. Secondly, in the signal splitter module, the power lines are routed to two branches, one of which directly connects to the sensor head module through its “SENSOR output” port, while the other acts as an external port to facilitate system expansion. Apart from the power distribution, the signal splitter module acts as a routing hub to the output of the sensor head module, wherein the sensor signal line is connected to its ground reference line. In order to improve the integrity of the signal as it travels over the long distance of the cables, a two-stage analog filtering network is used before the output signal with the “SIGNAL output” is taken to the external readout equipment. In the sensor head module, a piezoelectric disk acts as a sensor to convert the mechanical vibration into an electric signal. These raw signals, which are very weak, are directly fed into a high-impedance amplifier circuit, which is powered by the ± 12 VDC rails via the signal splitter. Additionally, the external port is used for further expansion, which is directly connected to the “SOURCE input” port of the additional signal splitter module.

**Fig. 3:**
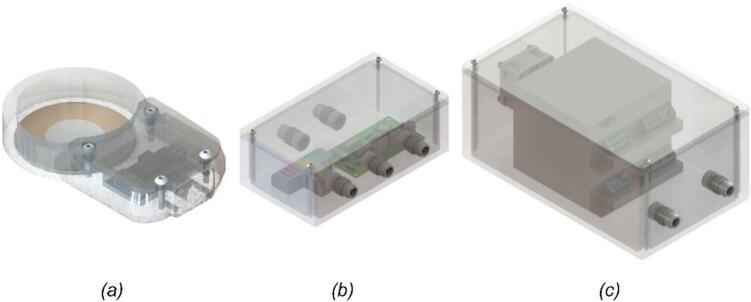
Transparent of the LUH modules: (a) the head sensor, (b) the signal splitter, and (c) the dual power.

Fig. 3 presents a transparent 3D model of the LUH, which displays the internal arrangement of the key components within the LUH enclosure. The transparent housing displays the space-saving arrangement of the LUH’s components. Components are arranged in the LUH so that they can be interconnected via wire connections and thus arranged flexibly according to the measurement location. Fig. 3 (a) shows the head sensor module with the piezoelectric disk arranged inside the housing and the amplifier board arranged close to the sensor to amplify the received infrasound signal before transmission to reduce noise and attenuation. Fig. 3 (b) depicts the signal splitter module, where the breadboard and filtering circuit are arranged at the bottom of the enclosure, and the connectors are organized to facilitate signal division as well as convenient wiring between modules. In Fig. 3 (c), the dual power module is displayed with its internal power supply units housed in a larger enclosure and external connectors provided for stable and flexible power distribution to the LUH system. From there, it shows a space-saving layout, and the flexible wire-based interconnection allows the modules to be conveniently arranged according to the measurement location.

**Fig. 4:**
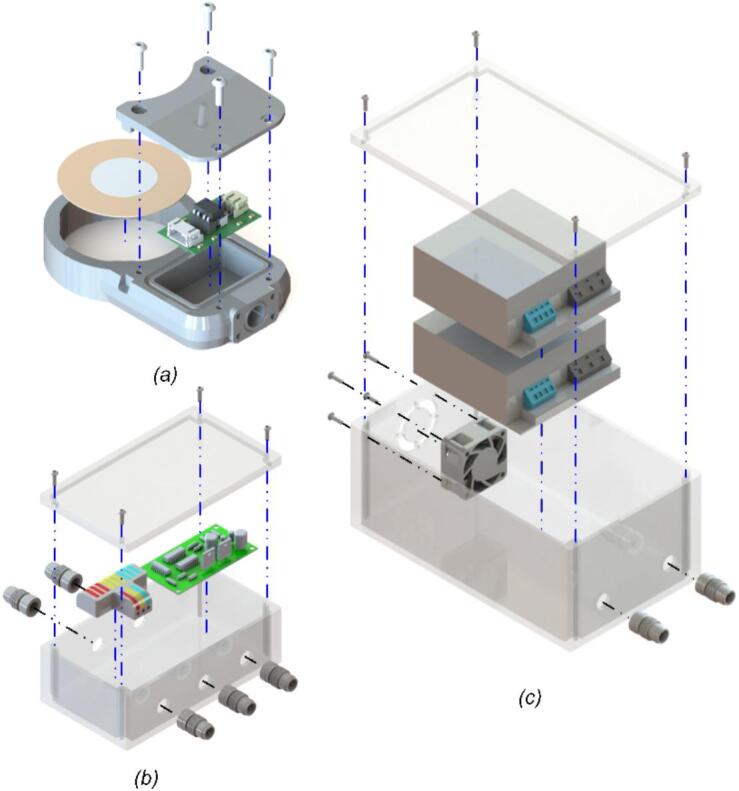
Installation of the LUH modules: (a) the head sensor, (b) the signal splitter, and (c) the dual power.

Fig. 4 depicts the installation of the three LUH modules. In general, the assembly principle of all modules employs a screwing mechanism to simplify the installation procedure. As shown in Fig. 4 (a), the head sensor module consists of a base with two compartments: a circular one for the piezoelectric disk and a rectangular one for the amplifier board. The piezoelectric disk is positioned using notches on the bottom edge, which allows the disc to be correctly aligned before the copper, silicone, and elastic dips are deposited. This is a very critical step, as once the dips have been deposited, no further adjustments can be made, and if the disc has been improperly aligned, the whole process has to be repeated using a different disc, which would result in time wastage. The rectangular compartment houses the amplifier board, which has the correct dimensions to fit the LPU, making it easy to place the board on the surface of the compartment. As shown in Fig. 4 (b) and Fig. 4 (c), the signal splitter module and the dual power module have a similar box-style enclosure, although the dimensions differ. Since neither of these modules has to be used underwater, the electrical components inside the enclosures are protected by securing the enclosure and the cover using screws, thus preventing exposure to dust or other environmental factors. In Fig. 4 (b), the breadboard is centrally positioned inside the splitter enclosure, with the pin header row arranged parallel to the sides and equipped with output connectors, enabling logical wiring and convenient observation for maintenance or repair. Finally, in Fig. 4 (c), the power module contains two stacked switching power supplies, complemented by a side-mounted fan that provides direct cooling to ensure stable operation.

**Fig. 5:**
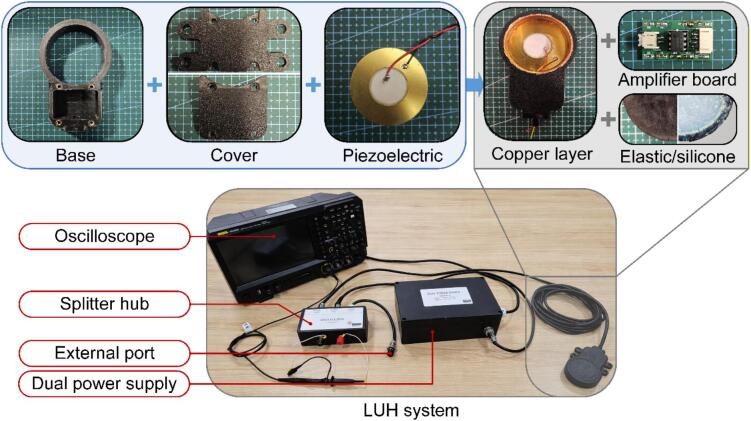
Complete modules after assembly.

Fig. 5 showcases the complete installation workflow of the LUH after being manufactured by 3D-printed technique with the surface refinement process, which aims for removing printing artifacts, as well as printed circuit board. The final 3D-printed products after being grinded and polished have a smooth substrate for subsequent epoxy filler application and spray coating. In addition to the sensor head module, the post-processing of the sensor base and the sensor coverage is performed by coating multiple layers of various materials for waterproofing and noise filtering. These layers include: (i) copper foil to form a conductive shielding layer, (ii) a 14 mm silicone layer to provide mechanical damping and waterproofing, and (iii) an outer 0.5 mm elastic dip coating to enhance water resistance and mechanical stability. In sequence, the completed modules after assembly are shown, in which each module is equipped with connectors to allow modular integration.

## 6 Operation instructions

**Fig. 6:**
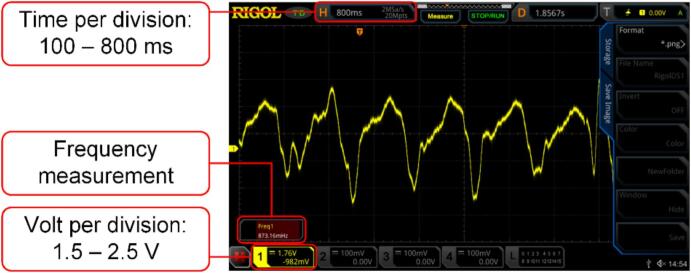
The oscilloscope user interface.

The oscilloscope consists of three principal areas: (1) the control and adjustment interface, (2) the display region, and (3) the input/output connectors. In the control and adjustment interface, there are vertical scaling knobs for voltage adjustment, horizontal time-base controls, trigger configuration buttons, and measurement function keys. Besides, the display region demonstrated in Fig. 6 provides real-time visualization of waveform behavior along with numerical measurement data: (i) time per division, (ii) mode of measurement, which normally turns into frequency measurement, and (iii) volts per division. In the lower and side panels, there are BNC input connectors, including channel 1 used to receive the hydrophone signal via coaxial cable. To observe the infrasonic signals, an appropriate configuration of the time base is necessary by adjusting the horizontal knob. Since a 20-Hz signal corresponds to a period of 50 ms, the horizontal time scale needs to reach a suitable value that allows one complete cycle to be displayed clearly. Typically, a setting between 100 ms/div and 800 ms/div enables visualization of one to three full cycles across the screen. Additionally, after being amplified, the voltage per division stays between 1.5 V and 2.5 V. If the waveform appears compressed on the horizontal axis, increasing the time-per-division setting stretches the waveform; conversely, decreasing the time-per-division setting. Sequentially, adjustments are conducted continuously until achieving a stable and interpretable sinusoidal pattern. On the other hand, the vertical scale is adjusted to ensure that the waveform occupies a substantial portion of the screen without clipping. Thus, verification of signal acquisition can be performed by alternately activating and deactivating the acoustic source, while changing the time-per-division and vertical scale. Upon activation of the excitation source is turned on, a periodic trigger waveform should appear immediately. Adjusting the time scale during this process further confirms that the displayed frequency exists trigger changes under the appropriate horizontal scaling. In the case of the non-existence of a trigger frequency, the hydrophone is supposed to fail to record the acoustic signal. Once correctly configured, the oscilloscope display provides multiple quantitative parameters, including the measured frequency in hertz, the corresponding signal period in milliseconds, peak-to-peak voltage, RMS voltage, maximum and minimum voltage values, mean voltage level, sampling rate, and trigger status. For a properly captured 20 Hz sinusoidal waveform, the displayed frequency should approximate 20 Hz with minimal deviation, the period should be close to 50 ms, and the mean voltage should remain near zero.

In addition, proper placement of the sensor head is important for reliable low-frequency underwater measurements. During operation, the sensor head should be fully submerged and mechanically positioned using its mounting holes, so that its sensing surface faces the acoustic source, and there are no displacements that can introduce low-frequency artifacts. To reduce the influence of reflections and unwanted disturbances, the sensor should be placed away from the tank wall and the water surface. Besides, the other modules, including the dual power supply module, signal splitter module, together with the external equipment, such as an oscilloscope, are kept on the ground near the testing environment, which is dependent on the length of the output signal cable of the sensor head module.

## 7 Validation and characterization

### 7.1 Case study 1 − Laboratory validation of infrasonic measurement capability

**Fig. 7:**
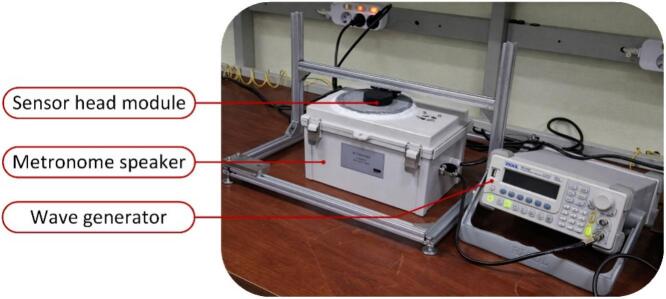
Experimental arrangement in the laboratory.

The first experiment is conducted in the laboratory environment to verify the ability of the LUH to detect infrasonic signals within the threshold of 20 Hz. This experiment is performed on the ground with the purpose of confirming the integrity of the LUH detection with the design of the piezoelectric sensing element, the high-impedance amplification stage, and the shielding structure, under repeatable and stable conditions. The experimental setup illustrated in Fig. 7 consists of a metronome speaker driven by a function generator configured to output a pulse waveform at 20 Hz. The sensor head module is mounted on a fixing frame, where the surface of the sensor head module is positioned in direct contact with the diaphragm of the speaker. This direct-contact configuration ensures the transmission of low-frequency oscillations to the piezoelectric element, reducing the possibility of energy loss.

**Fig. 8:**
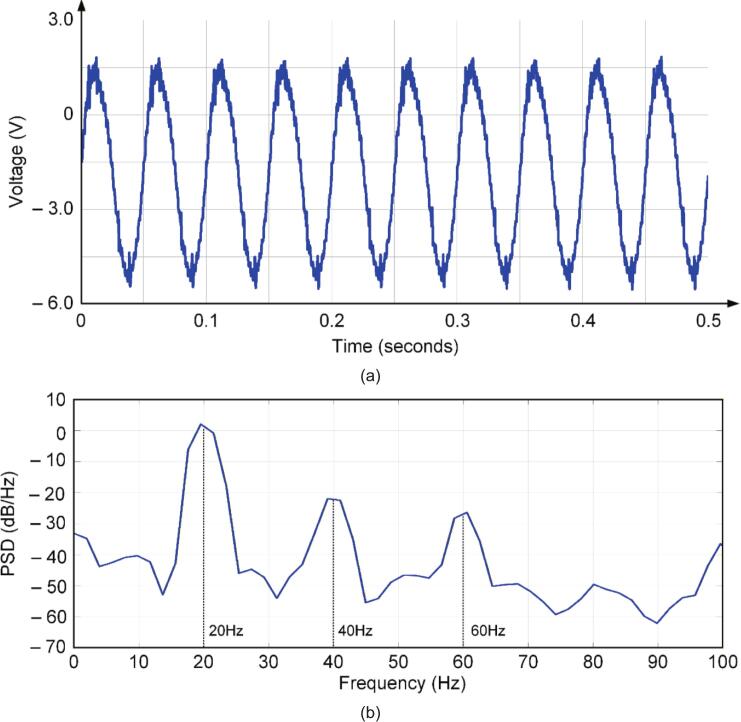
(a) Time-domain signal and (b) PSD analysis of 20 Hz data from a function generator detected by the LUH and captured using a 200 ms oscilloscope.

Fig. 8a illustrates the output signal measured in a laboratory setup using a 20 Hz signal generated by a function generator. The waveform exhibits a stable oscillatory pattern over the observation interval, indicating that the LUH system maintains consistent signal acquisition under the applied excitation. At the same time, the waveform is not purely sinusoidal and contains spike-like disturbances as well as sharp local peaks superimposed on the main oscillation. These features are attributed to the transient response of the excitation source, because the abrupt switching of the pulse signal generates mechanical impulses in the speaker diaphragm. The resulting impulses introduce additional vibration components that are transferred to the piezoelectric sensor, and this leads to localized distortions in the measured waveform. To evaluate the dominant frequency and the presence of other spectral components in the signal, the power spectral density (PSD) is analyzed as illustrated in Fig. 8b. The result shows a dominant peak at approximately 20 Hz, indicating that the main energy of the detected signal is concentrated at the intended excitation frequency. In addition, secondary spectral components appear at higher frequencies, particularly around 40 Hz and 60 Hz. These components are consistent with harmonic contributions and spectral spreading caused by the non-sinusoidal pulse excitation and its transient behavior. Although these additional components are present, their amplitudes remain significantly lower than that of the dominant peak. Overall, the PSD result confirms that the LUH system effectively captures the main low-frequency component, while the remaining spectral features are mainly associated with excitation-induced transients. The complete experimental procedure and demonstration are documented in the supplementary file LUH_Instruction.

### 7.2 Case study 2 − Certification testing at the Vietnam directorate for standards, metrology and quality

The second validation scenario is performed at the Directorate for Standards, Metrology and Quality under the Ministry of National Defense of Vietnam, with the primary purpose of obtaining independent verification and institutional certification for the LUH device. While Case Study 1 focuses on confirming fundamental functionality in the laboratory environment using in-house instrumentation, this certification test is designed to assess measurement reliability under a standardized setup and with higher-grade equipment provided by the Directorate. Thus, the evaluation serves as an external confirmation of the LUH’s capability to detect infrasonic signals with improved measurement confidence. The experimental setup is conducted on the ground and follows the same procedure as case study 1, with the Rohde & Schwarz RTB2004 digital oscilloscope provided by the Directorate for Standards, Metrology and Quality. This oscilloscope enables more precise waveform capture and improved measurement resolution compared to the laboratory oscilloscope, which ensures the reliability of the LUH device in detecting the infrasonic acoustic. In this test, the signal excitation frequency is set to 12 Hz with a sinusoidal waveform to demonstrate infrasonic measurement in a lower band and to match the Directorate’s test protocol.

**Fig. 9:**
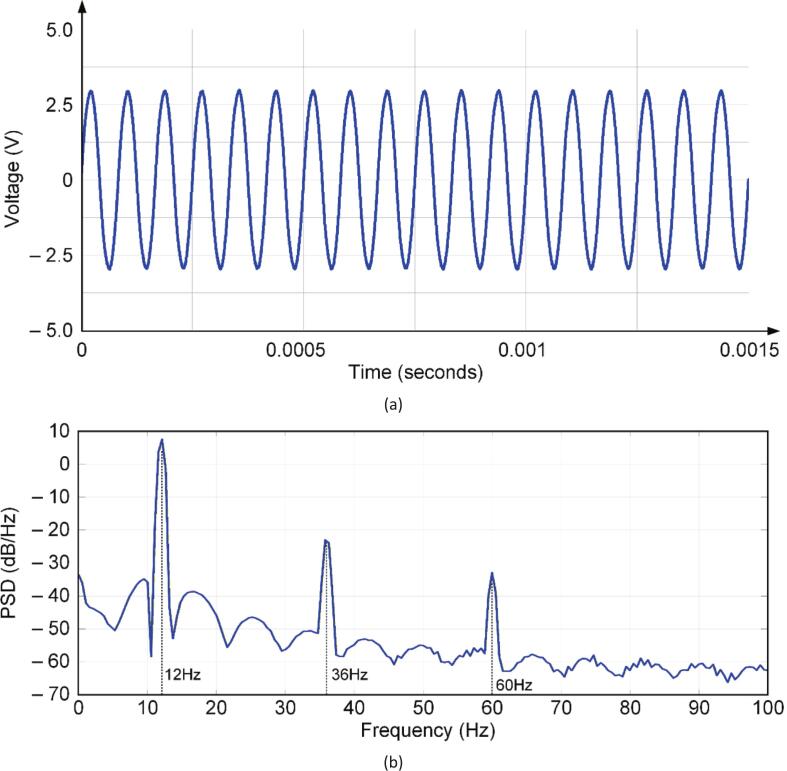
(a) Time-domain signal and (b) PSD analysis of 12 Hz data from a function generator as detected by the LUH and captured by a 190 ms oscilloscope provided by the Vietnam Directorate for Standards, Metrology and Quality.

Fig. 9a illustrates the measured output signal in the time domain under sinusoidal excitation at 12 Hz. The waveform exhibits a smooth and stable sinusoidal shape over the observation interval, indicating consistent signal acquisition and stable operation of the LUH system. The signal shows good symmetry between positive and negative cycles, while the amplitude remains nearly constant, confirming that the amplification stage operates without noticeable drift or low-frequency bias. In comparison with the previous case using pulse excitation, the waveform quality is significantly improved. The signal appears closer to an ideal sinusoidal form, with only minor irregularities observable near the peak regions. This improvement is attributed to the continuous nature of the sinusoidal excitation, which avoids abrupt transitions and reduces the generation of transient mechanical responses. As a result, the time-domain signal reflects a high-fidelity reconstruction of the input oscillation with minimal distortion. The spectral distribution of the measured signal is further examined through the PSD, as depicted in Fig. 9b. The energy is strongly concentrated around 12 Hz, forming a sharp and well-defined peak that corresponds to the excitation frequency. Beyond this primary component, several weaker spectral lines can be identified at higher frequencies, particularly near 36 Hz and 60 Hz. These features indicate the presence of harmonic content, which is commonly associated with slight nonlinearities in the excitation source and mechanical coupling path. Their comparatively low magnitude suggests that they do not significantly affect the representation of the main signal component. Briefly, the overall spectral profile demonstrates that the signal energy remains localized within the expected low-frequency region, while the remaining components are confined to minor contributions.

### 7.3 Case study 3 − Underwater frequency measurement of a biomimetic fish robot

**Fig. 10:**
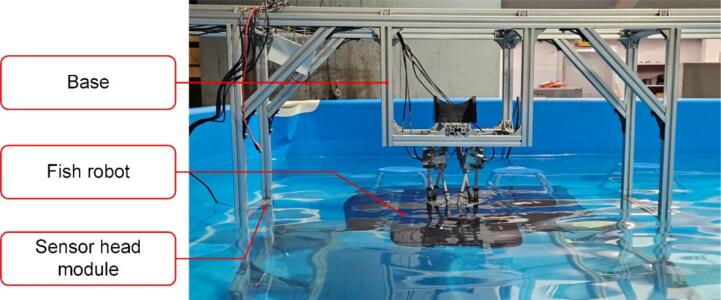
Experimental arrangement in the laboratory’s water tank.

The third validation scenario is to demonstrate a practical underwater environment, where the acoustic source is a complex electromechanical condition rather than a signal generator. The purpose of this case study is to assess whether the LUH is capable of detecting and characterizing the infrasonic emissions produced by a biomimetic fish robot developed within the lab. The biomimetic fish robot is intended for underwater exploration and environmental monitoring purposes. Consequently, it is required to operate at a low actuation frequency in order to avoid acoustic pollution that could disturb the aquatic life. In essence, the biomimetic fish robot utilizes a symmetric multi-propulsive fin (MPF) configuration and is propelled by a Central Pattern Generator (CPG) based on Hopf oscillators [20]. The CPG produces rhythmic control signals that generate periodic fin motions, and these motions induce pressure fluctuations and mechanical vibrations in the surrounding water, with the target operating frequency of 1 Hz. Nevertheless, the robot failed to integrate an independent acoustic sensing module to verify the actual emitted frequency. As a result, an external hydroacoustic measurement, the LUH device, is necessary to verify the real operating features of the fish robot’s actuation frequency. In this context, the LUH serves as a monitoring instrument to confirm whether the robot produces infrasonic emissions at the desired frequency. The experimental setup illustrated in Fig. 10 is implemented in the laboratory’s water tank, with the sensor head module submerged and positioned at a distance of approximately 0.3 m from the robot. This separation is chosen to reduce near-field effects such as turbulence and strong local flow disturbances. In addition, both the sensor head module and the robot are fixed using corresponding mounting frames, ensuring their relative positions remain constant during operation.

**Fig. 11:**
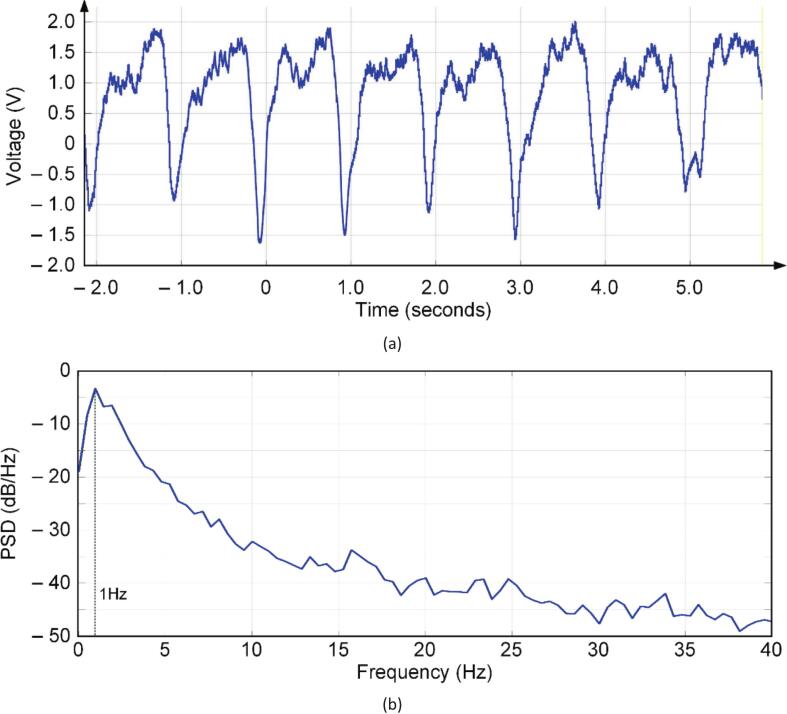
(a) Time-domain signal and (b) PSD analysis of 1 Hz data from a biomimetic fish robot detected by LUH.

Fig. 11a illustrates the measured signal generated by the biomimetic fish robot operating at a nominal frequency of 1 Hz. The waveform exhibits a repeating low-frequency pattern, confirming the presence of a periodic component associated with the robot’s actuation. However, the signal deviates from an ideal sinusoidal shape and presents noticeable amplitude variations and irregular fluctuations over time. These characteristics are attributed to the complex underwater environment, where hydrodynamic interactions, actuator nonlinearities, flow-induced disturbances affect both the robot motion and the resulting acoustic emission. As a result, the measured signal contains both the primary oscillatory behavior and superimposed disturbances, leading to a non-uniform waveform profile. Despite these variations, the periodic nature of the signal remains clearly observable, indicating that the LUH system can capture the actuation behavior of the robot under realistic operating conditions. Meanwhile, Fig. 11b presents the PSD of the measured signal, highlighting the distribution of energy across the frequency spectrum. The energy is primarily concentrated in the very low-frequency region, with a noticeable peak around 1 Hz corresponding to the actuation frequency of the robot. In contrast to the controlled excitation cases, the spectral profile appears more distributed, with energy gradually decreasing as frequency increases rather than forming distinct harmonic peaks. This behavior reflects the influence of hydrodynamic interactions, environmental noise, and the non-ideal motion of the robot, which introduce additional low-frequency components into the signal. Overall, the PSD confirms that the dominant signal content remains within the infrasonic range, while the surrounding spectral components represent secondary effects arising from the interaction between the robot and the fluid environment.

### 7.4 Capabilities and limitations of the LUH device

Based on the three validation scenarios, the LUH device demonstrates several practical capabilities for low-frequency underwater acoustic measurements, which are concluded:

• The LUH device is capable of detecting infrasonic signals under controlled laboratory excitation, as shown in the validation case study 1 and case study 2.
• The LUH can operate in a submerged environment and detect very low-frequency acoustic emissions of 1 Hz generated by the biomimetic fish robot.
• The modular design with low fabrication cost and usage of accessible components make the hardware suitable for research prototyping and educational activities in underwater acoustics.

At the same time, several limitations of the current LUH should be noted:

• The present validation was conducted only in laboratory-scale conditions, which does not confirm long-range performance or operation in open-water environments.
• The current study does not provide a complete validating characterization of the device, such as calibrated sensitivity, dynamic range, frequency response curve, or rigorous signal-to-noise ratio analysis.
• The waterproofing and shielding strategies were validated functionally during the reported experiments but were not evaluated through formal pressure testing or standardized electromagnetic compatibility measurements.

### 7.5 Conclusion

The study introduces an affordable open-source hydrophone device specific for infrasonic underwater acoustic measurements below 20 Hz. By combining 3D-printed structural components with a modular electronic architecture, the LUH device achieves a low total fabrication cost below 90 USD while maintaining the essential characteristics of low-frequency sensing. Besides, the design is modularized into the dual ± 12 VDC power supply module, the signal splitter module, and the sensor head module employing a piezoelectric element with a high-impedance amplification stage, with a standardized design guideline.

The performance of the LUH is validated by three case studies, including the laboratory experiment on the ground, the certification test by the Vietnam Directorate for Standards, Metrology, and Quality, as well as the underwater frequency measurement of the biomimetic fish robot. The first case study is the laboratory experiment on the ground, where the LUH device is able to detect the 20 Hz infrasonic signal under pulse waveform excitation. The second experiment, conducted independently at the Directorate for Standards, Metrology and Quality under the Ministry of National Defense of Vietnam, performs a highly clean and stable sinusoidal response at 12 Hz using high-precision instrumentation. The improved waveform and minimal noise level observed in this experiment support the LUH’s reliability with its institutional validation and certification. Finally, the third case study demonstrates the practical underwater application by measuring the infrasonic feature of a biomimetic fish robot operating at a target frequency of 1 Hz. Despite the increased complexity of underwater propagation and hydrodynamic disturbances from the surrounding environment, the LUH device detects the dominant 1-Hz-frequency component, enabling external verification of the robot’s operating frequency at a distance of approximately 0.3 m. In general, these experiments confirm that the LUH can reliably capture infrasonic acoustic signals below 20 Hz across both controlled laboratory conditions and realistic underwater scenarios. Nevertheless, several limitations remain, including the current validation experiments that are conducted at laboratory scale with functional assessments instead of overall calibration, and therefore provide only limited characterization of quantitative calibration, sensitivity, noise floor, uncertainty, and repeatability data; long-range underwater attenuation; and depth-dependent effects in natural environments.

In future work, field trials in open-water environments, studies on the influence of distance and depth, and overall calibration procedures will be prioritized. In particular, a fundamental metrological characterization of the sensor will be conducted, including the evaluation of key performance metrics such as sensitivity, signal-to-noise ratio, dynamic range, and frequency response using controlled excitation methods. Additional improvements may include integrating onboard digitization and data logging and developing standardized test protocols. Overall, this work demonstrates that a low-cost and validated infrasonic hydrophone can be realized using accessible fabrication methods and open-source design practices, providing a practical measurement for underwater acoustics research and education.

## Ethic statements

This work does not involve human participants, animals, or sensitive personal data. All experiments were conducted using laboratory equipment and engineered systems under controlled conditions. Therefore, no ethical approval or informed consent was required. Besides, the authors declare that no generative AI tools were used in the preparation of this manuscript.

## CRediT authorship contribution statement

**Nhut Thang Le:** Writing – original draft, Visualization, Software, Conceptualization. **Ngoc Hung Nguyen:** Writing – original draft, Visualization, Software, Data curation. **Cong Toai Truong:** Writing – original draft, Resources, Investigation, Conceptualization. **Huy Hung Nguyen:** Validation, Project administration, Formal analysis. **Tan Tien Nguyen:** Supervision, Funding acquisition. **Van Tu Duong:** Writing – review & editing, Methodology, Conceptualization.

## Declaration of competing interest

The authors declare that they have no known competing financial interests or personal relationships that could have appeared to influence the work reported in this paper.
